# Scalable solutions for global health: the SalivaDirect model

**DOI:** 10.3389/fcimb.2024.1446514

**Published:** 2024-10-29

**Authors:** Anne L. Wyllie, Brittany Choate, Laura Burke, Yasmine Ali

**Affiliations:** ^1^ Department of Global, Environmental, and Occupational Health, University of Maryland School of Public Health, College Park, MD, United States; ^2^ SalivaDirect, Inc., New Haven, CT, United States

**Keywords:** saliva, diagnostics, surveillance, innovation, pandemic, outbreak response, open-source, sustainable

## Abstract

The COVID-19 pandemic caught the world unprepared. Large-scale testing efforts were urgently needed, and diagnostic strategies had to rapidly evolve in response to unprecedented worldwide demand. However, the rollout of diagnostic testing and screening for SARS-CoV-2 was often impeded by logistical challenges, including regulatory delays, workforce shortages, laboratory bottlenecks, and supply chain disruptions. Recognizing these hurdles early on, we developed a testing approach that supported frequent, repeat testing, particularly as communities reopened. We hypothesized and experimentally demonstrated that saliva was a suitable specimen for the detection of SARS-CoV-2. This finding was advanced into the development of open-source, extraction-free reverse transcription polymerase chain reaction protocols using readily available, “off-the-shelf” reagents and equipment for the direct detection of SARS-CoV-2 in saliva (“SalivaDirect’’). Working with the US Food and Drug Administration (FDA), we established a novel regulatory framework wherein the FDA granted Emergency Use Authorization to Yale University to offer the SalivaDirect test protocol to high-complexity diagnostic laboratories (as designated by the Clinical Laboratory Improvement Amendments) with quality oversight provided by Yale. This grew into a network of more than 200 labs across the United States that, as of May 2024, resulted in over 6.5 million SARS-CoV-2 tests. By making the protocol flexible and open-source, laboratories were able to rapidly and economically scale testing using a simple, self-collected saliva specimen. Additionally, fostering a national network of laboratories enabled real-time exchanges, problem solving, and the development of community best practices. Preparing for the next pandemic, or simply the next seasonal epidemic, the SalivaDirect model of deploying a readily available, expandable solution and accompanying network provides a proven method for the successful implementation of pathogen testing in the United States and globally.

## Introduction

The COVID-19 pandemic caught the world unprepared to test populations for infection with SARS-CoV-2. From an epidemiological and infectious disease perspective, the emergence of SARS-CoV-2 as a highly infectious pathogen was not a question of if but when (Morens and Fauci, 2020). Since its initial emergence, SARS-CoV-2 variants have continued to evolve, each threatening diagnostic testing methods and therapeutic approaches.

Our global inability to rapidly roll out diagnostic testing and screening for SARS-CoV-2 was a result of inadequate supply chains, limited material availability, insufficient laboratory capacity, shortages of personal protective equipment (PPE), and a lack of clinical samples and standards for test validation. This placed unprecedented strain on healthcare personnel and necessitated assumptions to designate the appropriate sample type for testing. Based on established diagnostic practices for other respiratory infections, the nasopharyngeal swab was widely adopted as the recommended sample type for the detection of SARS-CoV-2. Consequently, early COVID-19 testing protocols often require nasopharyngeal specimens, which are invasive, require healthcare professional training for collection, and necessitate specialized swabs and stabilization medium. These factors contributed to patient discomfort, potential exposure risks for healthcare workers, and other important drawbacks such as testing aversion.

During the early COVID-19 pandemic, the COVID-19 response across Yale University was similarly confronted with significant supply chain and testing capacity issues. This forced our team to rethink our testing approach. Specifically, we (1) validated saliva as a sample type to overcome the challenges associated with the nasopharyngeal swab (Wyllie et al., 2020); (2) demonstrated the stability of SARS-CoV-2 in raw, unsupplemented saliva (Ott et al., 2021); (3) developed a simplified, open-source protocol using commonly available reagents and laboratory equipment, with saliva as the clinical specimen (Vogels et al., 2020a); (4) collaborated with the Food and Drug Administration (FDA) to obtain a novel Emergency Use Authorization (EUA) (SalivaDirect - EUA Summary); and (5) established a network of clinical laboratories across the United States (US), designated to use our streamlined polymerase chain reaction (PCR) test. What began as an urgent public health response has evolved into an innovative public health framework, providing a scalable model adaptable for future global health challenges.

## Saliva as a specimen of choice

Although not a traditional upper respiratory tract diagnostic sample type, saliva emerged early during the pandemic as a viable sample type for the detection of SARS-CoV-2 (Tan et al., 2021). Initial comparisons between swab-based and saliva specimens were conflicting, largely due to a lack of standardized collection and processing methods (Tan et al., 2021). Today, substantial evidence supports the equivalence of swab-based and saliva specimens for SARS-CoV-2 molecular testing in both symptomatic and asymptomatic individuals (Tobik et al., 2022). Additionally, studies indicate that SARS-CoV-2 RNA can be more abundant in saliva during the early stages of infection compared to nasal specimens (Adamson et al., 2022; Lai et al., 2022; Savela et al., 2022). Importantly, studies have also demonstrated that the detection of SARS-CoV-2 remains stable in raw, unsupplemented saliva for extended periods days to weeks and at elevated temperatures (Ott et al., 2021). As such, saliva has proven to be a reliable and sensitive specimen for SARS-CoV-2 diagnostic testing and screening protocols (Tobik et al., 2022). Moreover, saliva collection is noninvasive and can be reliably self-collected without trained personnel (Allicock et al., 2022), alleviating supply chain demands (e.g., no swabs, reduced PPE), permitting more affordable and frequent testing, lessening test aversion (Van de Casteele et al., 2022), and reducing exposure risk to healthcare personnel compared to swab-based methods.

## The SalivaDirect protocol

**Figure 1:**
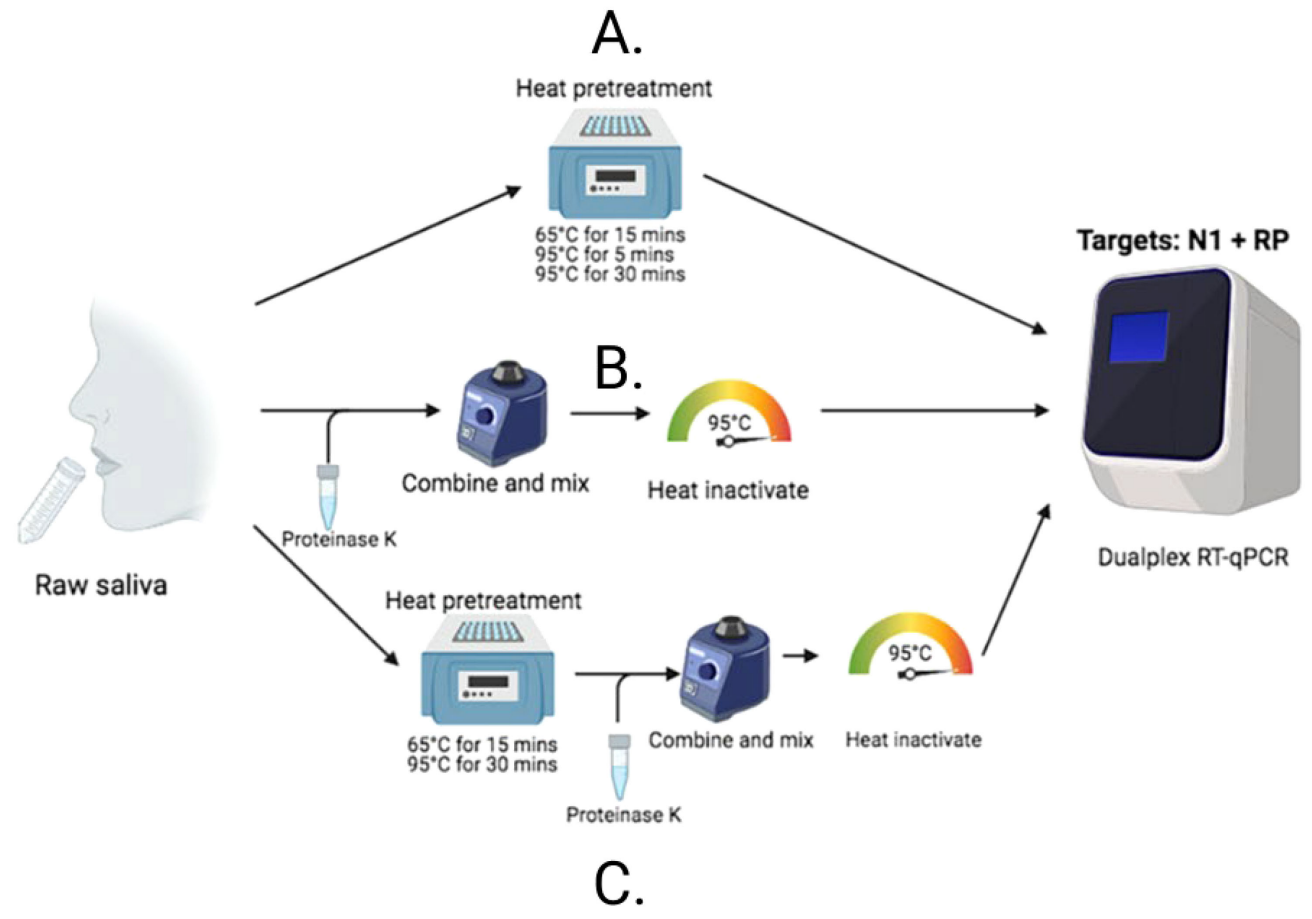
The SalivaDirect PCR protocol tests lysed saliva samples through one of three different workflows. **(B)** For Workflow 1, proteinase K is added to 50 uL of saliva which is vortexed vigorously to mix before heating at 95oC for 5 minutes to inactivate the proteinase K prior to testing in qPCR. **(A)** Workflow 2 requires only incubation of the saliva at one of three time and temperatures indicated before testing in qPCR. **(C)** Workflow 3 combines Workflows 1 and 2; the saliva is first heated to inactive virus before addition of proteinase K to further break down the sample for ease of PCR testing.

**Figure 2:**
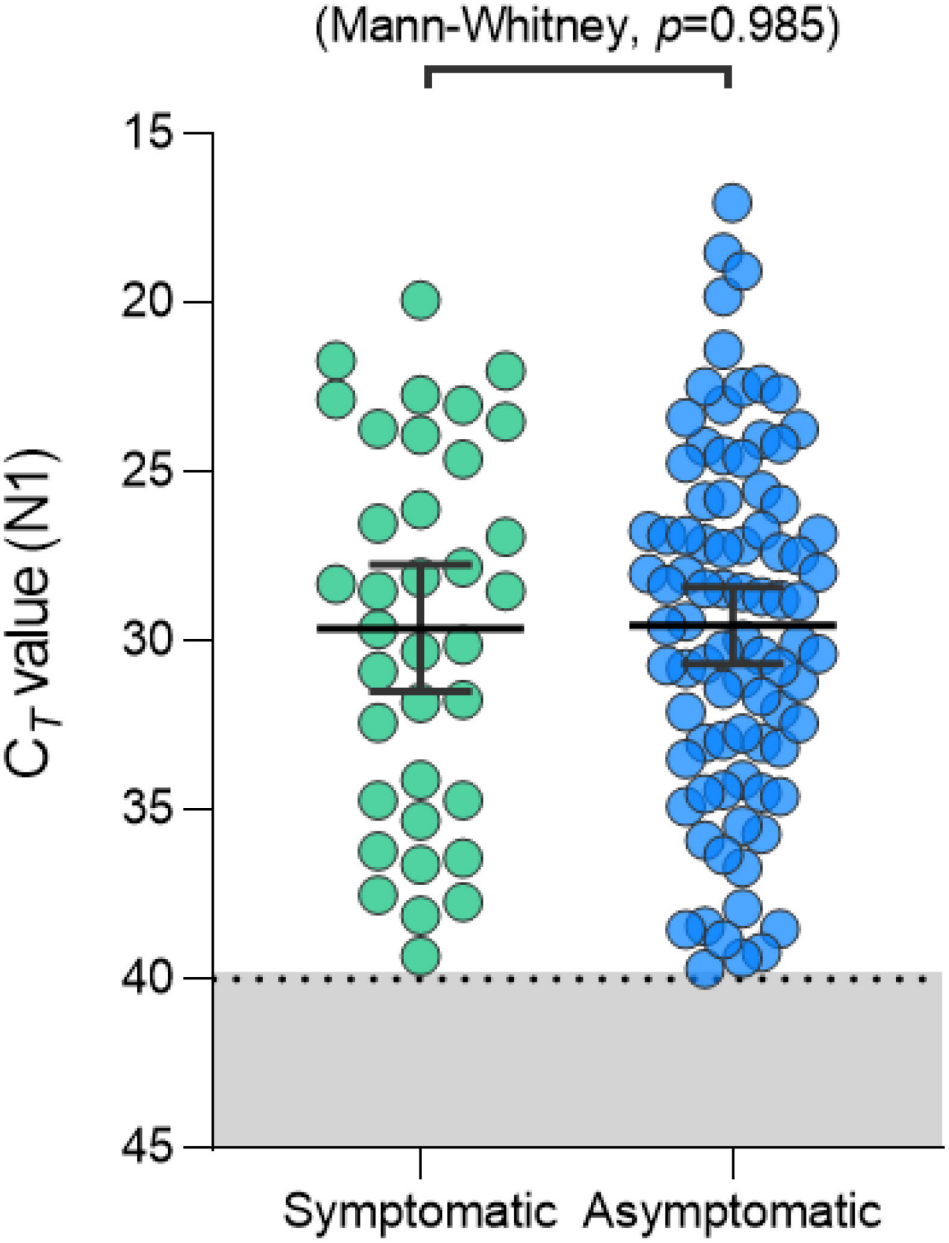
PCR Ct-values generated from saliva samples from symptomatic and asymptomatic COVID-19 patients were comparable. Saliva samples from 85 asymptomatic COVID-19 patients were tested using the SalivaDirect PCR protocol (workflow 1). Resulting Ct values were compared to those obtained from 36 symptomatic patients collected during the same period and also tested using the SalivaDirect PCR protocol (workflow 1). We found that the Ct values did not differ between patient groups, demonstrating comparable viral load despite symptom profile and that the probability of “missing” viral infection in asymptomatic individuals due to low viral loads was low.

After identifying that saliva performed comparably to, and sometimes better than, nasopharyngeal swabs for the detection of SARS-CoV-2 (Wyllie et al., 2020), and recognizing the barriers to testing that were often faced early in the pandemic, we were motivated to enhance access to testing by simplifying testing approaches. COVID-19 testing primarily relied on RNA extraction prior to molecular assay amplification, a cumbersome, time-consuming, and resource-intensive process. Thus, inspired by a study originally published in April 2020, assessing the direct (RNA extraction free) testing of nasopharyngeal swabs (Bruce et al., 2020), we explored its applicability to saliva. Using CDC-recommended reverse transcription polymerase chain reaction (RT-PCR) primer sequences and a standardized RT-PCR assay (Vogels et al., 2020b), we tested saliva with and without (direct) RNA extraction. Following mixed, albeit promising, results, we made additional modifications and found proteinase K treatment of native saliva followed by heating at 95°C (to inactivate the proteinase K) provided equal sensitivities (similar RT-PCR cycle threshold values compared to RNA extracted samples) (Vogels et al., 2020a). We validated this approach using a hospital cohort, comparing the detection of SARS-CoV-2 in saliva tested using this extraction-free PCR protocol and in nasopharyngeal swabs with a commercial RT-qPCR kit requiring RNA extraction; we observed a high positive agreement (94%) (Vogels et al., 2020a). Thus, the SalivaDirect protocol emerged (**Figure 1**) and on a large study cohort was subsequently demonstrated to detect asymptomatic and/or pre-symptomatic cases (**Figure 2**), with a low invalid and false positive test rate (Vogels et al., 2020a). Taken together, these findings indicated that the SalivaDirect protocol would be appropriate in a screening use case setting.

Our primary objective was to ensure the ease and affordability of implementing the SalivaDirect protocol in clinical laboratories. Unlike traditional clinical molecular assays, which often require proprietary testing kits or expensive specialized equipment, we developed the SalivaDirect protocol using an open-source approach. Our aim was to enable clinical laboratories to procure reagents and supplies from numerous vendors, thereby circumventing significant supply chain disruptions and leveraging their existing supplier relationships. Furthermore, we designed the protocol to be compatible with PCR instruments commonly found in most laboratories. Based on the list pricing of reagents and supplies from various suppliers, we estimated the cost per SalivaDirect test to range from $1.21 to $4.39 (Vogels et al., 2020a).

## A novel regulatory framework

Working with the US FDA, we established a groundbreaking regulatory framework. The FDA granted an EUA to Yale University, but in a first, delegated administration and oversight of the protocol to us, enabling the rapid deployment of the SalivaDirect protocol to qualified, Clinical Laboratory Improvement Amendments (CLIA)-certified high-complexity labs across the US. Interested labs were required to validate the protocol’s performance on an authorized PCR instrument and using validated reagents. Designated labs were required to adhere to the Instructions for Use and report all testing performance data to Yale, which analyzed and reported the data to the FDA.

To support the successful implementation of the SalivaDirect protocol, a collaborative approach was imperative. We worked individually with each lab to help them through the onboarding process. We built a network for labs to connect to share the required validation materials when necessary or to develop assay proficiency testing protocols. Hosting regular videoconferences, message boards, and manning a highly responsive email account, we listened to the specific needs of both interested and designated labs to build out a more flexible list of equipment and reagents in our protocol through amendments to our EUA.

**Figure 3:**
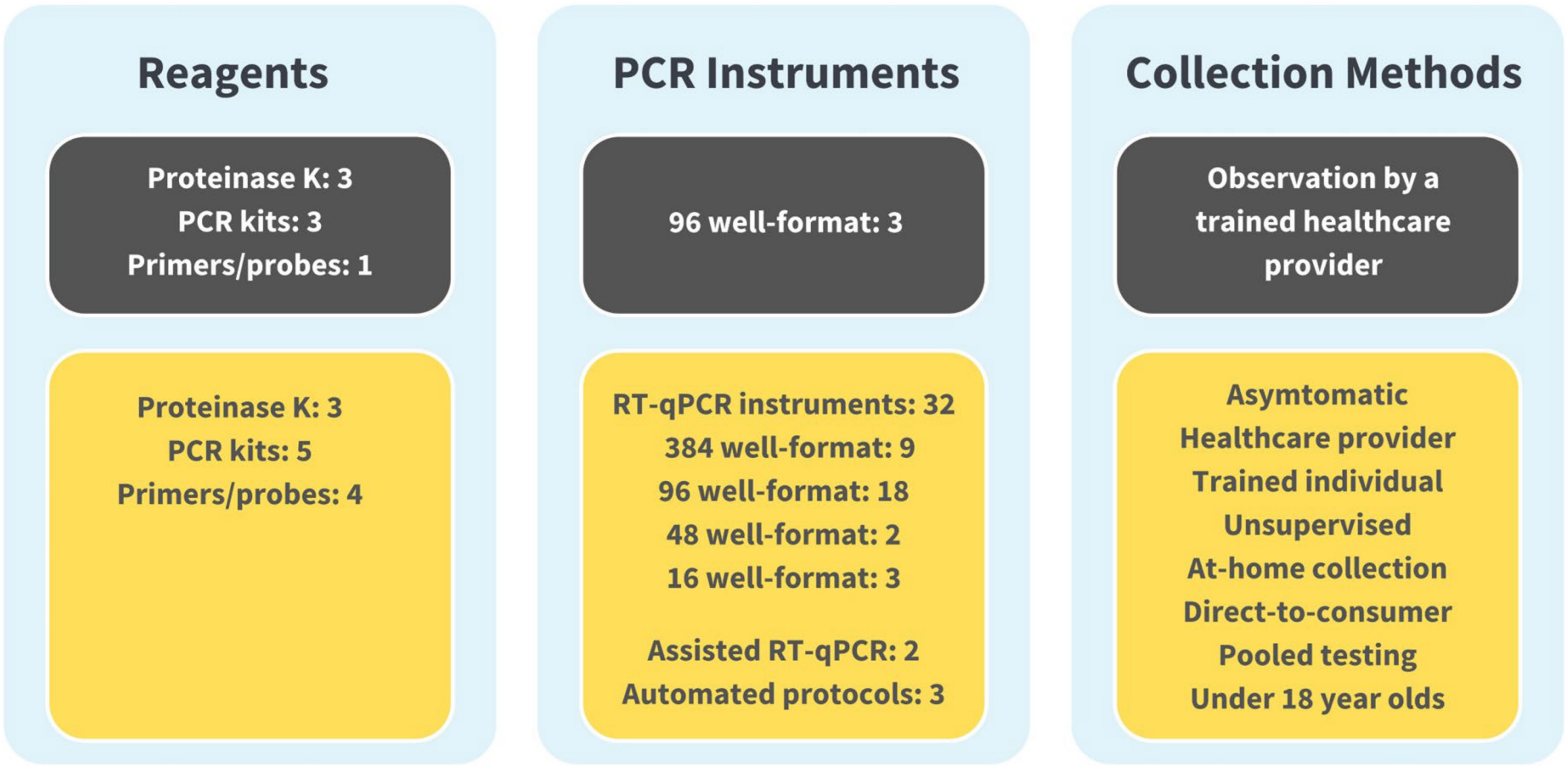
The SalivaDirect Emergency Use Authorization (EUA), now and then. For broad adoption, we expanded our original EUA protocol (shown in grey boxes)l to include a wider range of workflows (**Figure 1**) and polymerase chain reaction (PCR) instruments (shown in yellow boxes). By expanding our protocol to include equipment and supplies commonly found in labs, labs could adopt the SalivaDirect PCR protocol and operate under our EUA without significant financial investments in new equipment. Being able to source reagents from multiple suppliers helped to overcome supply chain disruptions while providing options for pricing. Broad collection methods facilitated testing options, permitting sample collection from the clinic to schools and workplaces to the home setting.

The evolution of the SalivaDirect protocol from initial EUA to June 2024 can be seen in **Figure 3**. Over 25 amendments have been submitted for reasons including supporting the broader adoption of the protocol, high-throughput testing (addition of automated protocols), or permitting the inclusion of additional materials to support lower cost testing and avoid supply chain issues. These amendments were accomplished through bridging studies that established equivalent performance between parallel testing of saliva specimens with new and previously validated protocol components. Currently, SalivaDirect, Inc. holds four EUAs, including provisions for direct-to-consumer and at-home collection methods and continues to expand the use of SalivaDirect to fill evolving and future needs.

To better establish true performance and compare different assays, the FDA developed reference materials to establish an absolute limit of detection for each authorized assay (Office of the Commissioner, 2020). We participated in this post-authorization validation using our least sensitive combination of reagents and equipment. Compared to other FDA SARS-CoV-2 RT-PCR tests with EUA, this SalivaDirect workflow had a limit of detection comparable to or better than most manufactured PCR assays for swabs with RNA extraction (range = 540–540,000 units/mL). Our measured limit of detection with the FDA reference material was 18,000 detectable units/mL (MacKay et al., 2020).

SalivaDirect continues to be focused on regulatory strategies that allow for great flexibility while maintaining rigorous quality standards to address barriers to non-invasive testing. Our mission continues to be the expansion of equitable testing by continuing to pursue full FDA clearance for the SalivaDirect protocol and developing new molecular assays for the detection of influenza virus, respiratory syncytial virus (RSV), and other respiratory pathogens of concern (Allicock et al., 2023a). We continue to seek innovative solutions to testing accessibility through initiatives like our mobile testing van, the expansion of collection sites to include vending machines, and to pursue regulatory approval for these collection methods. SalivaDirect was successfully deployed to more than 200 labs across 42 states with a range of over 20 instruments and to date has recorded more than 6.5 million tests with a false positive/negative rate of less than 0.01%.

## Non-supervised self-collected saliva

Our original SalivaDirect EUA was awarded on the provision that saliva collection was observed by trained healthcare personnel, yet the workforce was limited, and this increased the cost of testing. To broaden the utilization of saliva collection, we first worked with the FDA to create a protocol under which any individuals could become trained to observe the collection of saliva for testing using SalivaDirect. To further expand access to testing, we subsequently demonstrated that saliva could be reliably “self” collected in a non-supervised manner (Allicock et al., 2022). We recognized the importance of clear instructions for the collection of good-quality saliva samples; we developed a comprehensive collection protocol to guide non-technical individuals through self-collecting an appropriate saliva specimen, whether on-site or at-home. Importantly, we demonstrated that the detection of SARS-CoV-2 remained stable in raw (unsupplemented) saliva in the absence of cold-chain (Ott et al., 2021) to further reduce the burden of collection and transport of samples for testing.

Upon the authorization of these non-supervised protocols by the FDA, these collection instructions were provided to the laboratory network for broad implementation. In line with our dedication to protocol flexibility, these collection protocols included a broad list of validated components. Both network labs and outside manufacturers were permitted to create saliva collection kits based on these protocols, which could then be distributed within each lab’s specimen collection network, removing a barrier to saliva collection and enabling significant scaling of SARS-CoV-2 testing.

## Scaling SalivaDirect for mass testing

Reopening the US economy, schools, workplaces, and sports leagues required the implementation of mass SARS-CoV-2 screening and surveillance programs with frequent testing to quickly identify and isolate infected individuals, thereby mitigating transmission. For these efforts to be sustainable, we recognized that testing approaches needed to reduce costs, resources, healthcare worker involvement, and PPE usage; support sample collection and return at convenient locations; easily integrate into established laboratory accessioning and pre-analytic workflows; and provide acceptable clinical sensitivities and specificities. While the SalivaDirect method met each of these criteria, pooling samples promised a more economical and higher throughput solution for screening. However, labs expressed significant concerns about the logistical burden of implementing pooling and its potential negative impact on sensitivity (Fenichel et al., 2021).

With this in mind, we developed and validated a series of pooling workflows (Allicock et al., 2023b) to provide laboratories additional cost-saving flexibility. All workflows, validated for pools of five samples, demonstrated adequate sensitivity, achieving 98% positive agreement for detecting SARS-CoV-2 compared to individual sample testing. While most labs found the low per sample cost of SalivaDirect sufficient to continue with individual sample testing, a few labs adopted the pooling approach to further reduce costs and increase sample throughput.

## SalivaDirect network

**Figure 4:**
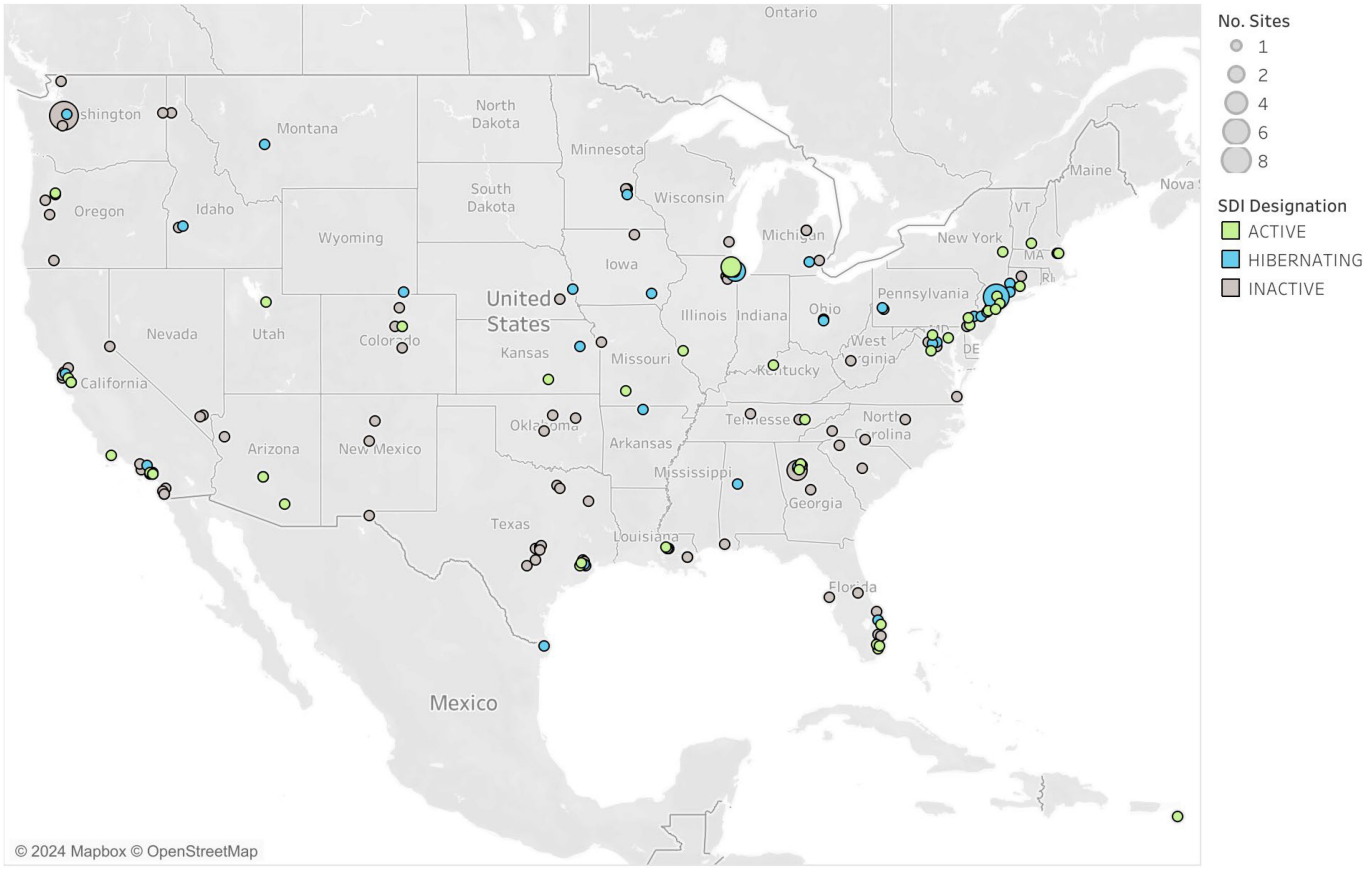
Distribution and status of SalivaDirect designated clinical laboratories across the US.

Since August 2020, more than 200 high-complexity CLIA-certified laboratories across 42 US states and territories have been designated by Yale University to test using the SalivaDirect PCR protocols (**Figure 4**). Collectively, as of May 2024, over 6.5 million SARS-CoV-2 tests have been recorded with reported false positives and negatives accounting for less than 0.01% of tests. Labs have been crucial in the development of new methods for testing, serving as study sites, conducting collaborative research, and piloting new programs. Testing capacity for the network remains high (over 133,000 per day) even as demand for COVID-19 testing is falling, indicating the laboratory network stands ready when the need for surge testing arises.

The network of labs within the SalivaDirect network highlights the versatility of the protocol with its implementation in a variety of qualified sites. SalivaDirect enrolled traditional hospital-based or public health labs that implemented SARS-CoV-2 testing alongside their typical diagnostic services (e.g., testing for inpatients, admissions, and/or community-based programs). Many labs emerged from universities or in support of professional organizations who, almost overnight, built out laboratory infrastructure and obtained CLIA certification to serve their own populations and often, their broader communities. Still, other labs arose from private entities which opened their doors to accommodate the exorbitant testing burden placed on the healthcare system. Many of these labs were set up using readily available PCR instruments, circumventing the backlog of equipment manufacturer delays. Their mandate was to scale as quickly as possible and provide screening services to students, faculty, and workers, often providing 2,000-30,000 SalivaDirect test results per week. Laboratories used the SalivaDirect protocol to complete testing in a plethora of high-risk communal settings with vulnerable populations such as daycare centers (Rayack et al., 2023), K-12 schools (Mendoza et al., 2021; Virji, 2021), university campuses (De Santi et al., 2021; Vander Schaaf Nicole et al., 2021), prisons, and to support high-profile gatherings such as sports leagues and Broadway (Adamson et al., 2022).

In mid-2021, network labs were surveyed about their reasons for adopting the SalivaDirect protocol and joining the network. Although responses varied, specific themes emerged. The first theme centered on reagents. The ability to choose reagents from various sources, obviating supply chain disruptions and providing low cost of goods was highly desirable. The second driver was being able to provide COVID-19 testing using a molecular assay with EUA. Of note, the labs highlighted that they felt comfortable working with SalivaDirect to obtain their designation and appreciated the availability of straightforward RT-PCR assay protocols that were easy to understand and implement, even by those with little experience in adopting and performing open-source assays. The third reason for adoption was the saliva specimen type itself. Most, if not all, labs had experienced issues with nasal-based specimens, either due to supply chain constraints, the collection method itself, or healthcare provider shortages and safety concerns. The fourth and final theme was appreciation for the network itself. Member labs found that the open dialog and technical guidance provided by the SalivaDirect team, during group video calls or one-on-one conversations, facilitated adoption, and subsequent scaling of testing. In 2024, laboratories continue to support the SalivaDirect initiative, looking forward to the development of multiplex protocols and planned clinical studies to address public health concerns.

Since 2020, the SalivaDirect framework with its unique community network has had a favorable impact on SARS-CoV-2 testing, be it (a)symptomatic diagnosis or screening. We have shown that simple testing protocols can be centrally developed, meet regulatory requirements, and be mobilized by a large group of extremely different yet highly motivated high-complexity CLIA-certified labs to address evolving public health needs. We showed that it is possible to establish a standardized testing process that can be applied across multiple sites in different regions, and even different countries (López-Martínez et al., 2020; de Oliveira et al., 2021; De Santi et al., 2021; Rodríguez Flores et al., 2021), which is a strategy that can be used to improve future data quality and for surveillance purposes.

Use of the protocol and an active lab network continues to exist, even with the slowdown of COVID-19 testing. By maintaining network partnerships, this highly skilled group of “pandemic” experienced clinical laboratories and scientists can continue to build on the successes seen during the pandemic and be ready to rapidly introduce new tests, scale testing on a need-by-need basis, and impactfully address changing public health needs. We continue to provide technical support, invite opportunities for collaboration, and crowd-source suggestions for future diagnostic targets. Current network engagement efforts are led by SalivaDirect, Inc., a 501c(3) public health nonprofit that spun out from Yale University in 2023 and is working to develop and deploy innovative diagnostic solutions that improve global public health. SalivaDirect, Inc. continues to maintain and build network connections through a range of activities from organizing international webinars to leveraging the network to obtain saliva specimens for the validation of new test protocols for identifying additional infectious diseases or chronic conditions. Beyond this, SalivaDirect, Inc. actively works with communities to deploy innovative solutions, including the development of mobile testing programs that remove barriers to access and deliver diagnostic options directly to traditionally underserved populations.

## SalivaDirect: a model for outbreak response and pandemic preparedness

Looking ahead, the SalivaDirect model, from its flexible protocol to network engagement, holds significant potential for rapidly responding to future pandemics (Fleming et al., 2021; Dixson-Declève et al., 2023). The opportunity for remote, non-observed saliva collection provides a mechanism for broadening access to testing. During the COVID-19 pandemic, we demonstrated the value of open-source protocols, which can be rapidly adopted and scaled through a diverse, CLIA-certified high-complexity laboratory network.

In preparing for the next public health challenge, scalability of testing to meet specific communities is paramount. This requires broader access to reagents and supplies beyond typical diagnostic supply chains, utilization of simple collection options like saliva, and the availability of easy-to-perform protocols that allow for laboratory flexibility and sample pooling. These factors were significant drivers of scalability and rapid assay adoption during the COVID-19 pandemic.

A growing body of literature demonstrates that viral and bacterial respiratory pathogens can be detected in saliva with clinical sensitivities similar to those of nasal specimens (Laxton et al., 2023). Furthermore, we and others have shown that the SalivaDirect method, or similar extraction-free approaches, can be adapted to detect other respiratory pathogens such influenza A/B, RSV, human metapneumovirus (Allicock et al., 2023a), mpox virus (Thomas et al., 2023), pneumococcus (Peno et al., 2023), and Group A *Streptococcus (* Peachey et al., 2024 *).* These findings suggest immense potential for rapidly modifying the SalivaDirect method and disseminating new diagnostic tests throughout the SalivaDirect network, facilitating swift and effective responses to emerging infectious threats.

Beyond the test protocol, the SalivaDirect network of labs provides a fundamentally important structure for collaboration and education between labs during pandemic or public health challenges. Network labs offer coordinated testing capabilities across the US, catering to diverse demographics and needs. Moreover, they can serve as indicators or sentinels to identify and quantify pathogen spread or the emergence of new variants, crucial for effective healthcare responses.

## Conclusion

Through the establishment of a unique regulatory framework, the SalivaDirect model produced an impactful pandemic response, increasing access to SARS-CoV-2 testing by bringing together a diverse network of CLIA-certified high-complexity labs. Key to the rapid adoption and network growth was innovation in the regulatory space that utilizes robust quality controls by the developer, collaboration with the FDA, and harnesses the expertise of laboratory scientists to enable the provision of open-source, rigorously validated protocols that were not limited to single vendor reagents or instruments. We have demonstrated that this model can facilitate implementation of broad screening and diagnostic molecular testing for an emerging respiratory pathogen and, as a result, should be considered as we prepare for future outbreaks or the emergence of new global health challenges. Moreover, our pragmatic approach holds great potential to address challenges faced by resource-limited communities (Tan et al., 2022; Pai et al., 2023). By offering cost-effective, easily deployable solutions suitable for decentralized sample collection, the SalivaDirect approach presents a practical option for addressing evolving global testing needs. As we contemplate future pandemic preparedness, the SalivaDirect model of innovation and collaboration offers valuable insights into scalable, adaptable testing strategies. Its success suggests that it could serve as a blueprint for global health initiatives seeking to enhance testing accessibility, efficiency, and effectiveness. By building upon the lessons learned from SalivaDirect, we can fortify our collective readiness to confront the public health challenges of tomorrow.

## Data Availability

The raw data supporting the conclusions of this article will be made available by the authors, without undue reservation.
